# Ganoderma lucidum methanolic extract as a potent phytoconstituent: characterization, in-vitro antimicrobial and cytotoxic activity

**DOI:** 10.1038/s41598-023-44135-1

**Published:** 2023-10-13

**Authors:** Seyyed Mojtaba Mousavi, Seyyed Alireza Hashemi, Ahmad Gholami, Navid Omidifar, Wei-Hung Chiang, Vijayakameswara Rao Neralla, Khadije Yousefi, Mansoureh Shokripour

**Affiliations:** 1 Department of Chemical Engineering, National Taiwan University of Science and Technology, Taipei, Taiwan; 2 Health Policy Research Center, Health Institute, Shiraz University of Medica Sciences, Shiraz, Iran; 3 Biotechnology Research Center, Shiraz University of Medical Sciences, P.O. Box: 71348-14336, Shiraz, Iran; 4 Pharmaceutical Sciences Research Center, Shiraz University of Medical Sciences, Shiraz, Iran; 5 Department of Pathology, School of Medicine, Shiraz University of Medical Sciences, Shiraz, Iran

**Keywords:** Biotechnology, Microbiology, Plant sciences

## Abstract

*Ganoderma lucidum methanolic extract* (*GLME)* has attracted tremendous attention due to its exceptional antimicrobial and anticancer properties that can be delicately tuned by controlling the initial extraction's content and concentration. Herein, we detailed the characterization, antimicrobial, and cytotoxic performance of *GLME* as a potential multi-functional therapeutic agent. Accordingly, FTIR, XRD, FESEM, EDX, and HPLC analyses were employed to assess the samples, followed by disc diffusion and microdilution broth methods to test its antibacterial effects against four Gram-positive and Gram-negative bacterial strains, viz., *Enterococcus faecalis*, *Staphylococcus aureus, Escherichia coli, and Pseudomonas aeruginosa*. MTT assay was applied to determine the cytotoxic activity of *GLME* against PDL and Hek-293 normal cell lines and MCF-7 and K-562 cancer cell lines. The IC50 values of 598 µg mL^-1^ and 291 µg mL^-1^ were obtained for MCF-7 and K-562 cancer cell lines, which confirmed the stronger anticancer activity of the *GLME* against blood cancer cells than breast cancer cells. This is while the IC50 of normal Hek-293 cells is 751 µg mL^-1^, and the lowest toxicity was observed for normal PDL cells with more than 57% survival at a concentration of 3000 µg mL^-1^. The results showed that the antibacterial property of this product against *E.coli* bacteria was higher than streptomycin, so the zone of inhibition was observed as 44 ± 0.09 mm and 30 ± 0.11 mm, respectively. These data provide valuable insights into the therapeutic usage of *GLME* for treating breast and blood cancers. This work is motivated by research studies looking for pharmacological products to address chronic and acute diseases, where further resources and studies are required to explore such products' adverse effects and toxicity.

## Introduction

A species of medicinal fungus namely Ganoderma lucidum methanolic extract (*GLME*) has been utilized for many years in Chinese medicine. It has been found to possess various therapeutic properties, including anti-inflammatory, antioxidant, and immune-boosting effects. The remarkable medicinal and pharmacological potential of (*GLME*) has captured the interest of researchers and scientists alike. This is primarily attributed to the mushroom's abundance of significant pharmacologically active compounds^1–3^. *GLME* is enriched with diverse biologically active compounds, including proteins, triterpene, amino acids, polysaccharides, vitamins, enzymes, flavonoids, steroids, alkaloids, and minerals. Due to their significant pharmacological properties, polysaccharides and triterpenoids are the main focus of research regarding *GLME*. Over the past four decades, researchers have identified 431 secondary metabolites, over 380 terpenoids, and more than 30 compounds from the group of steroids in various Ganoderma species^4,5^. The presence of polysaccharides and triterpenoids in *GLME*’s structure has numerous pharmacological features. Subsequently, many experiments have been accomplished to investigate the performance of this natural product against different types of cancers like prostate cancer^6^, lung cancer^7^, colon cancer^8^, and cervical cancer^9^. Also, *GLME*’s other pharmacological characteristics, such as anti-inflammatory^10^, hypoglycaemic^11^, hypocholesterolemic^12^, antioxidant activity^13^, cardio-protective, hepato-protective, and anti-allergic activity, have been evaluated by researchers^1^.

Estrogen receptor-positive breast cancer, such as MCF-7 cells, accounts for a significant proportion of all breast cancers^14,15^. Leukemia is a complex disease that can be classified into different types depending on the type of blood cells affected and the speed of disease progression. Chronic myeloid leukemia, from which K-562 cells are derived, is a slow-growing form of leukemia that affects the myeloid cells in the blood and bone marrow^16,17^. The lack of effective treatment options for advanced stages of cancer is a major contributing factor to the high mortality rate associated with cancer^18,19^. Several therapeutic approaches have been approved for treating this condition regardless of the disease's stage. However, because malignant tissue is dynamic and heterogeneous, the effectiveness of particular conventional treatments is dwindling^20,21^.

Triterpenoids and polysaccharides derived from *GLME* have also been shown to display anticancer effects through a variety of mechanisms. The anticancer properties of immune cells are activated by the *GLME* polysaccharides, which stimulate the immune system (B cells, T cells, and macrophages,) to create cytokines^22,23^. In addition to boosting the immune system, polysaccharides have been shown to have anticancer effects through a variety of mechanisms, including cytotoxicity, decreased integrin expression to prevent tumor cell adhesion, promotion of cancer cell apoptosis, and inhibition of angiogenesis^5,24,25^. *GLME* polysaccharides with anticancer potential include lengthy glycosidic linkages and high molecular weights^22^. It has been suggested that the anticancer activity of these polysaccharides is due to their ability to bind to complement receptor type 3, which is responsible for their interaction with branched β-1-3- and β-1-6-d-glucans^5,26^. High molecular weight anticancer substances include glycoproteins (heteropolysaccharides) and ganoderan A-B-C^5^. According to studies, *GLME* triterpenoids have anticancer properties that include decreasing cancer cell proliferation and assault and blocking protein kinase C or catenin activity^1,23,27^.

It has been demonstrated that triterpenoids, particularly ganoderic acids, exert cytotoxicity in a variety of cancer cells^26^. There have been reports of the ganoderic acids, ganoderic acid F, ganoderic acid T, and ganoderiol D having potent anticancer action^1^. Furthermore, it has been demonstrated that Y, U, X, W, and V ganoderic acids have cytotoxic effects on hepatoma cells^26^. Additionally, *GLME* has beneficial bioactive substances with immunomodulatory and anticancer activities that can operate as natural resources to lessen the side effects of conventional chemotherapy and/or radiation and boost the immune systems of cancer patients^1^. Therefore, this research was planned and carried out in two parts to determine the characterization of *GLME* and investigate this natural product's antibacterial and cytotoxic activity. In the first part, to enhance *GLME’s* properties, sample extraction was done via the standard method. The disc diffusion assay was used to test *GLME*'s antibacterial tolerance against *Staphylococcus aureus*, *E. coli*, *Enterococcus faecalis*, and *Pseudomonas aeruginosa*. The microdilution broth approach was then used to determine this substance's minimum inhibitory concentrations (MICs) and minimum bactericidal concentrations (MBCs), with both bactericidal and bacteriostatic effects defined. In the final section of this investigation, the cytotoxic activity of *GLME* against human breast cancer cells and human blood cancer cells was assessed through MTT assay.

## Materials and methods

### Sample extraction and preparation

Pure-dried *GLME* was purchased from a traditional market in Shiraz, Iran. The dried *GLME* was ground to form a fine powder, and then its essence was extracted by combining it into a tube with methanol at room temperature. In this regard, 2.5 g of dried *GLME* was first blended with 100 mL of methanol and then shaken for 24 h at 125 rpm. In the next step, the mixture was filtered using filter paper and placed in an oven at 50 °C for 6 h. Subsequently, 3 mg of the final extracted essence was dissolved in DMSO as a stock solution for biological assays. As an alternative, the stock solution was diluted in PBS or culture medium before use for in vitro treatments. The DMSO concentration in the final product never surpassed 0.2 vol.%.

### GC/MS analysis

To analyze *Ganoderma lucidum (GL)*, we added 100 g of powder to 3 mL of distilled water and collected the oil through hydrodistillation into hexane. Following this, the solution was concentrated at room temperature and the essential oil was analyzed using a gas chromatograph (model HP 6890) and mass spectrometer (model 5973) from Agilent Technologies. We used an HP-5 MS capillary column (5% phenylmethylsiloxane (30.0 m × 250 μm × 0.25 μm)) and helium as a carrier gas, starting with a column temperature of 120 °C for 5 min. Then, the column temperature was increased from 5 °C per minute to 320 °C and it was held for 5 min. We performed electron impact ionization for mass spectroscopy at an ionization energy of 70 eV. We automated the injection of 2 mL of the diluted sample into the mass spectrometer after diluting the essential oil with 98% hexane. Using Chem-Office software linked to the MS library, we were able to identify the constituent chemicals. The database of the National Institute of Standards and Technology was used to determine their structures, weights, molecular formulas, and names^28^.

### Characterization

*Escherichia coli* (ATCC 25922), *Staphylococcus aureus* (ATCC 29213*), Pseudomonas aeruginosa* (ATCC 27853), and *Enterococcus faecalis* (ATCC 29737) were purchased from Persian Type Culture Collection. Periodontal ligament (PDL) fibroblasts were purchased from the Sivan (Shiraz, Iran) Company. The Hek-293, MCF-7, and K-562 cells were purchased from (Pasteur Institute, Tehran, Iran). Different analyses were carried out to test the properties of *GLME*. FTIR spectroscopy with KBr tablets (Bruker model Tensor II) and X-ray diffraction (XRD) (Panalytical model X'Pert Pro, Almelo, Netherlands) was used to explore its crystallinity. The morphology of *GLME* was studied by a field emission-scanning electron microscope (FESEM, Tescan model Mira III, Brno, Czech Republic) equipped with an energy dispersive spectroscopy (EDX). An Azura HPLC device (Knauer, Berlin, Germany) fitted with a quaternary gradient pump unit and a UV–vis detector (190–700 nm) were used to study the contents of monosaccharides and disaccharides from *GLME*. The detector's wavelength was set to 250 nm. The components were separated by a Knauer C18 column (4.6 mm, 250 mm i.d., 5 m). Solvents A (acetonitrile) and B (benzene) made up the mobile phase (0.045 wt% KH_2_PO_4_), where the flow rate and injection volume were set on 0.8 mL min^-1^ and 20 mL, respectively. The mobile process was screened with a 0.45 mm filter and degassed under vacuum until further use. The system was run at ambient temperature.

### Disc diffusion assay

To prepare the sample microorganisms, standard *Enterococcus faecalis*, *Staphylococcus aureus, Escherichia coli, and Pseudomonas aeruginosa* strain were incubated at 37 °C for 24 h and then diluted in normal saline to achieve a concentration of 0.1 M. A spectrophotometer was used to read the optical density (OD) at 600 nm. After that, the disc containing soaked and saturated *GLME*, the disc containing streptomycin as a positive control, and the disc containing 2% DMSO as a negative control were placed on the solid surface of Muller-Hinton agar. Plates were placed within the incubator at 37 °C for 18 h, and finally, the diameter of inhibition zones was measured^29–31^.

### Minimum inhibitory concentrations (MICs) assay

90 µL of Mueller–Hinton broth (Merck, Germany) was added to the wells of the 96-well plates. Then, 90 µL of *GLME* at a concentration of 1000 µg mL^-1^ was added to the first well, and after the contents of the well were mixed, 90 µL was removed from the first well and added to the second well. This procedure was continued until the last well, and finally, 90 μL was discarded from the last well. In the next step, 10 μL of 600 nm OD (0.5 McFarland) microorganisms were transferred into the mentioned wells containing the developed samples. The plates were then incubated for 24 h at 37 °C. The final stage involved measuring the optical density of each well at a wavelength of 600 nm (BioTek, Power WaveXS2) and comparing results with a blank value and the positive control. As a positive control, streptomycin (1 mg mL^-1^ in sterile physiological saline) was utilized. The negative control was 2 vol.% DMSO. This procedure was carried out in triplicate.

### Minimum bactericidal concentrations (MBCs) assay

To determine MBC, the broth was taken from each well and incubated in Mueller Hinton Agar at 37 °C for 24 h for bacteria. In related tests, streptomycin acted as a positive control. The MBC was established as the lowest concentration of the necessary *GLME*, resulting in the total eradication of the incubated microbe^9,13^. The concentration of each compound causing no growth of microorganisms was regarded as the MBC^32^. Each test was performed in triplicate.

### MTT assay

PDL, Hek-293, MCF-7, and K-562 cell lines were used to test the cytotoxicity of *GLME*. The positive control was 5-Fluorouracil and carboplatin. After cultured cells in the T-75 flask, the cells were counted and seeded on 96-well plates with approximately 10,000 cells per well containing DMEM culture media and incubated to achieve 85 to 90% confluence. The previous media was then used to substitute 100 µL of *GLME* in a wide variety of concentrations. Next, in each well, 30 mL of MTT (3-(4,5 Dimethylthiazol-2-yl)-2,5-diphenyltetrazolium) stock solution (concentration 4 mg mL^-1^) was transferred and incubated under normal conditions. Purple formazan crystals formed due to the viable cells' mitochondrial function, and we used 100 µL of dimethyl sulfoxide (DMSO) to dissolve these crystals. The plate was shaken in a double orbital manner (for 5 min) to dissolve formazan crystals completely. Finally, the optical absorption of the mentioned solution was recorded at 540 nm using an Elisa plate reader (Model 50, Bio-Rad Corp, Hercules, California, USA)^33,34^. All tests were accomplished in triplicate^35^. The following equation describes the calculations of the cell viability:

$$\% \,Cell\,\,viability = \frac{[OD(cell + compound) - OD(compound)]}{{[OD(cell) - OD(Culture\,\,media)]}}*100$$

### Statistical analysis

The statistical interpretations in this analysis were made using the Statistical Package of the Social Sciences (SPSS) 22 software (SPSS Inc., Chicago, IL, USA). The antibacterial and cytotoxic activities of *GLME* were compared using one-way ANOVA/Tukey experiments. After three trial replications, the significance amount was set at 0.05.

## Results and discussion

### Characterization

**Figure 1:**
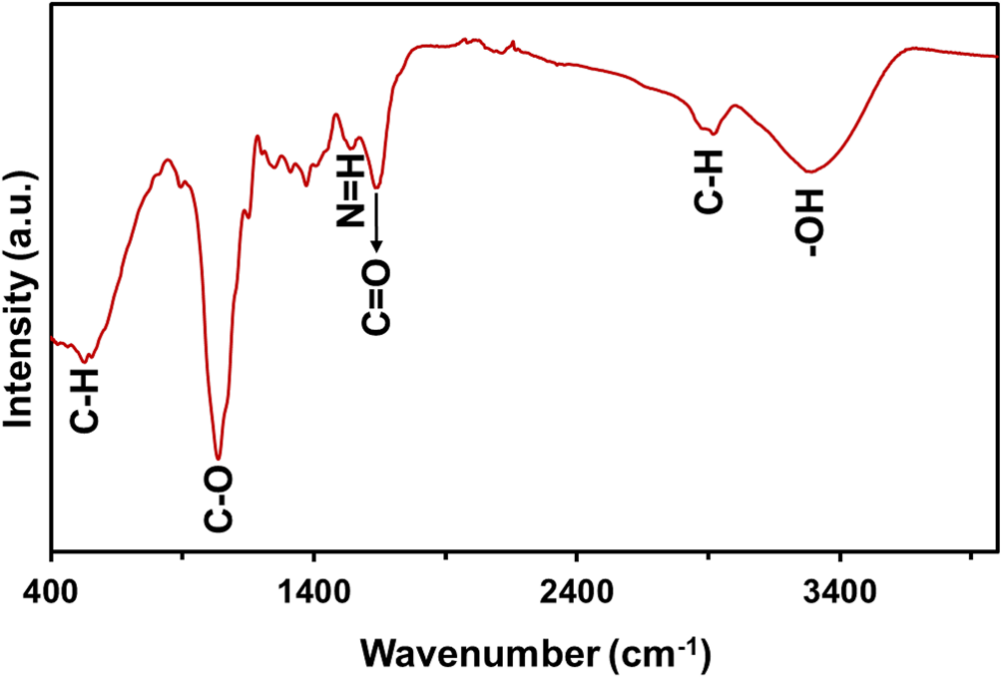
FTIR spectrum of *GLME.*

FTIR anlysis of *GLME was performed using *a KBr tablet in the spectrum range of 400–4000 cm^−1^. As demonstrated in Figure 1, the narrow peak at 2919 cm^−1^correspond to the C–H stretching vibration in aliphatic compounds, while the peaks at 1981 cm^−1^ and 3292 cm^−1^ showcases the presence of aromatic and hydroxyl functional groups (-OH), respectively 1. For *GLME*, several transmittance bands in the regions of distinct amide bands suggest the presence of proteins^36^*.* The stretching frequency of the C=O groups is associated with the amide-I band (1634 cm^−1^), while the bending vibration of the N=H groups is associated with the amide-II band (1538 cm^−1^). *GLME* had a critical characteristic band of 1035 cm^−1^, close to the stretching of the C–O bond. This banding pattern can also be seen in other Chinese medicines, including *Radix achyranthes, Cordyceps bidentatae*, and *Radix cyathulae*^37^. According to Barker et al.^20^, the 893 cm^−1^ band in the fingerprint area of d-glucopyranose is one of the most significant recorded bands. In sugar, this band is a C-H bending vibration. The bending vibration of a saccharide group is represented by the bands at 554 cm^−1^ and 529 cm^−1^^38^.

**Figure 2:**
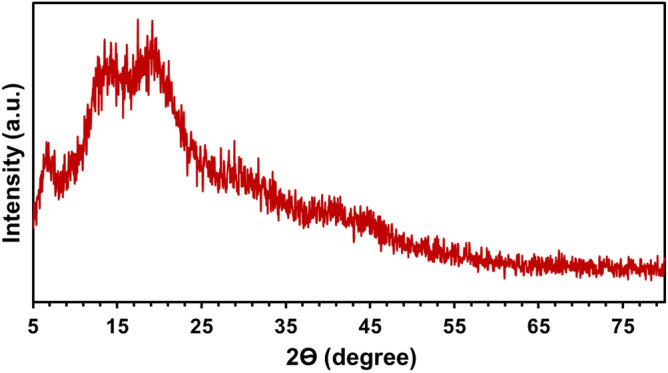
XRD pattern of *GLME.*

The X-ray diffractogram is employed to assess the degree of crystallinity of *GLME*; the obtained X-ray diffractogram can be seen in Fig. 2. As illustrated the XRD pattern showcased an amorphous compound with a broad peak at about 2Θ of 20°. No other peaks existed at higher scattering angles ^39^.

**Figure 3:**
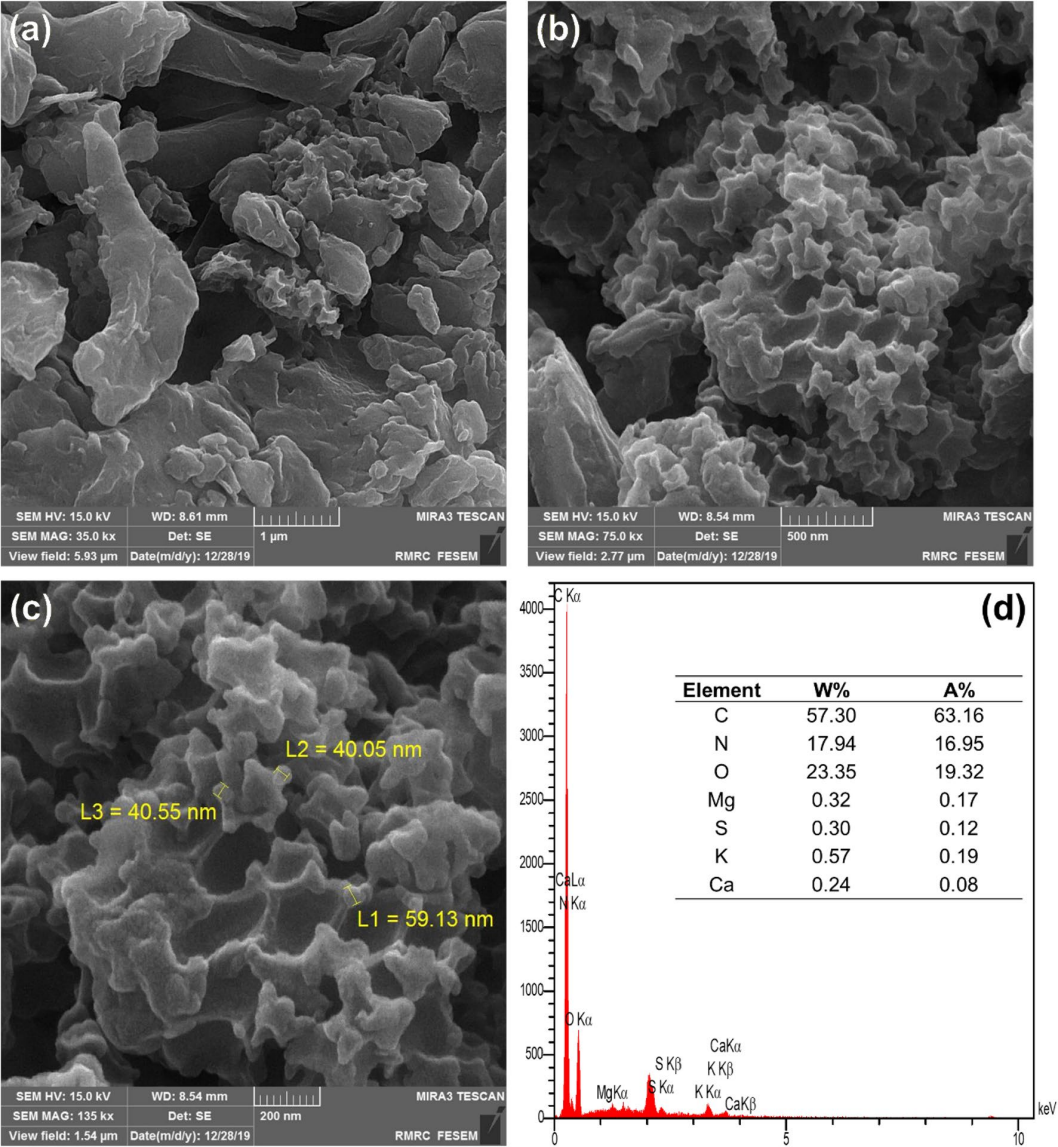
FESEM images and EDX analysis of the obtained nanoparticles from the *GLME*.

Fig. 3 illustrated the FESEM images of the extracted nano-sized particles from the *GLME* at different scales (i.e., 1 mm, 500 nm, and 200 nm) plus thier corresponding EDX scan. *GLME* showcased an amorphous morphology with particle sizes less than 60 nm. The obtained FESEM images showed that the *GLME* particles were ground into nanosized powders by the milling process. Declining the size of obtained particles from the *GLME* to nano-size could improve the yield during the essence extraction. This natural commodity also contained significant amounts of carbon, nitrogen, oxygen, magnesium, sulfur, potassium, and calcium, according to EDAX analysis (see Fig. 3).

Gas chromatograms were used to analyze the composition of GL, revealing the presence of various compounds with their respective retention times (RT) measured in minutes. The detected compounds include pentadecanoic acid, 14-methyl-ester (RT = 19.46 min; comprising 12.87% of the total content), hexadecanoic acid, ethyl ester (RT = 19.907 min; 7.45%), (Z, Z)-9,12-octadecadienoic acid methyl ester (RT = 20.865 min; 22.11%), and octadecanoic acid (RT = 22.737 min; 33.24%). These four oils make up a significant portion of the total oil content in *GL*, accounting for 75.67%. Additionally, there are three minor components present: l-Ascorbic acid 2,6-dihexadecanoate, (E)-9-Octadecanoic acid, methyl ester, and (Z)-9-Octadecenamide (RT = 19.554 min, 21.631 min, and 27.48 min; comprising 2.12%, 1.29%, and 1.88%, respectively). These minor components contribute to a total of 5.29% of the content in *GL* (Table 1).

**Table 1:** GC/MS analysis of *GLME.*

Peak number	Compound	Retention time	Area (%)	Molecular weight
1	Hexanoic acid	8.482	0.25	116.16
2	Nonanoic acid	10.633	0.09	158.24
3	Pentadecanoic acid	17.941	0.21	242.40
4	l-Ascorbic acid 2,6-dihexadecanoate	19.554	2.12	652.9
5	pentadecanoic acid, 14-methyl-ester	19.46	12.87	270.45
6	Hexadecanoic acid, ethyl ester	19.907	7.45	284.55
7	(Z, Z)-9,12 Octadecadienoic acid methyl ester	20.865	22.11	294.47
8	Heptadecane, 2, 6, 10, 15 Tetramethyl	20.95	1.66	296.12
9	(E)-9-Octadecanoic acid, methyl ester	21.631	1.29	296.48
10	Octadecanoic acid	22.737	33.24	284.47
12	Carbamic acid, 2-(dimethylamino)ethyl ester	27.391	1.21	132.16
13	(Z)-9-Octadecenamide	27.48	1.88	281.48
14	Hexadecanoic acid, 2-hydroxy-1-(hydroxymethyl)ethyl ester	28.331	0.21	330.50
15	6,9-Octadecadienoic acid, methyl ester	30.876	0.41	294.50
16	(Z)-9-Octadecene, 1-[2-(octadecyloxy)ethoxy]	31.052	1.55	565.0

**Figure 4:**
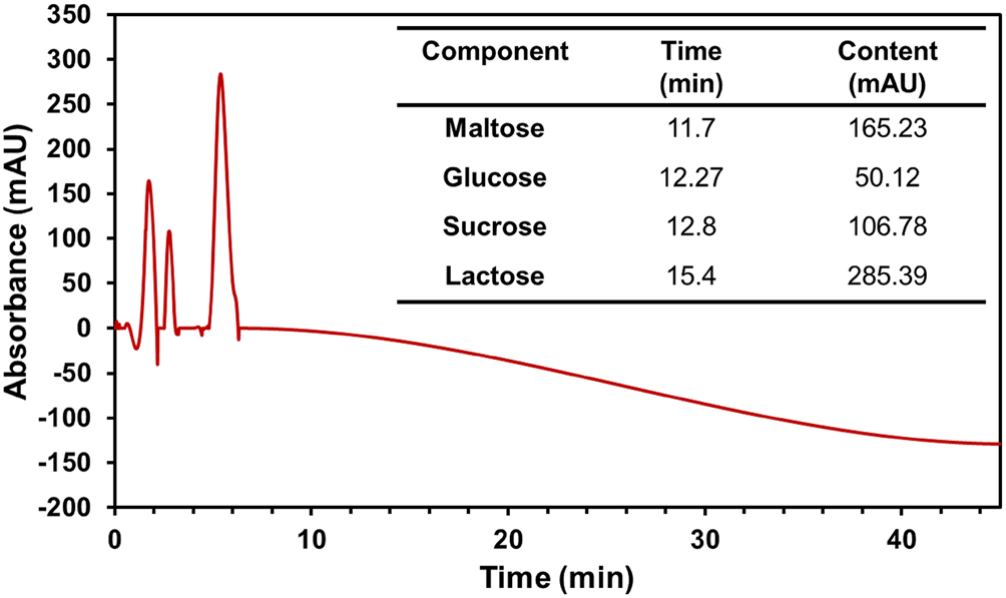
HPLC analysis of the *GLME.*

The contents of monosaccharides and disaccharides from *GLME* were calculated using an HPLC analysis in optimum separation conditions, with acetonitrile-0.045 wt% KH_2_PO_4_ as the mobile step and a flow rate of 0.8 mL min^-1^ at 250 nm as the detector wavelength. Polysaccharides from *GLME* were detected by comparing the retention time of each part with standard curves. Monosaccharides and disaccharides, such as lactose, glucose, sucrose, and maltose, were defined in Fig. 4.

### Cytotoxic study

**Figure 5:**
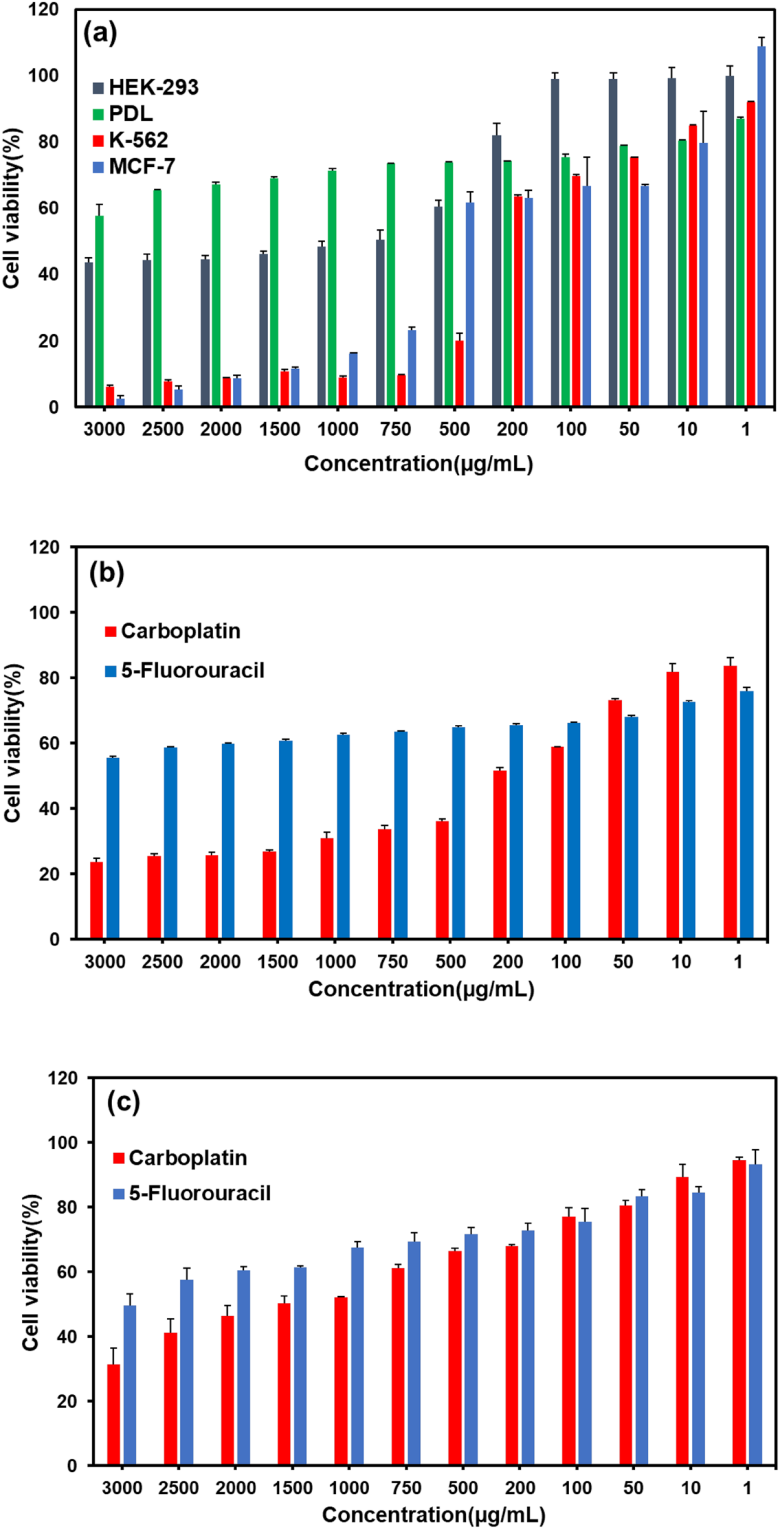
(**a**) Effects of methanolic *GLME* extraction on normal cell lines PDL, Hek-293, and cancerous MCF-7 and K-562 cells. Effects of Carboplatin and Fluorouracil anticancer drugs on (**b**) Hek-293 and (**c**) MCF-7 cell lines. The results are interpreted by finding the mean and standard deviation of the three separate experiments.

**Figure 6:**
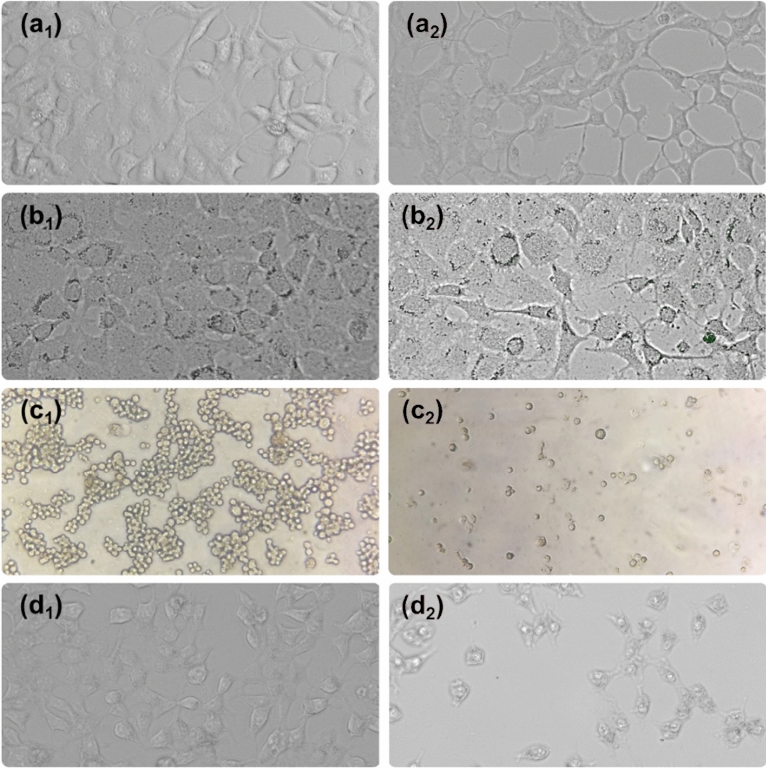
The morphological changes of cancer and normal cells (control cells (**a**_**1**_) Hek-293, (**b**_**1**_) PDL, (**c**_**1**_) K-562 and (**d**_**1**_) MCF-7) and treatment with *GLME* (treated cells (**a**_**2**_) Hek-293, (**b**_**2**_) PDL, (**c**_**2**_) K-562, and (**d**_**2**_) MCF-7) after 12 h at a concentration of 3000 µg mL^-1^ at a temperature of 37 °C with a humidity of 95%.

**Figure 7:**
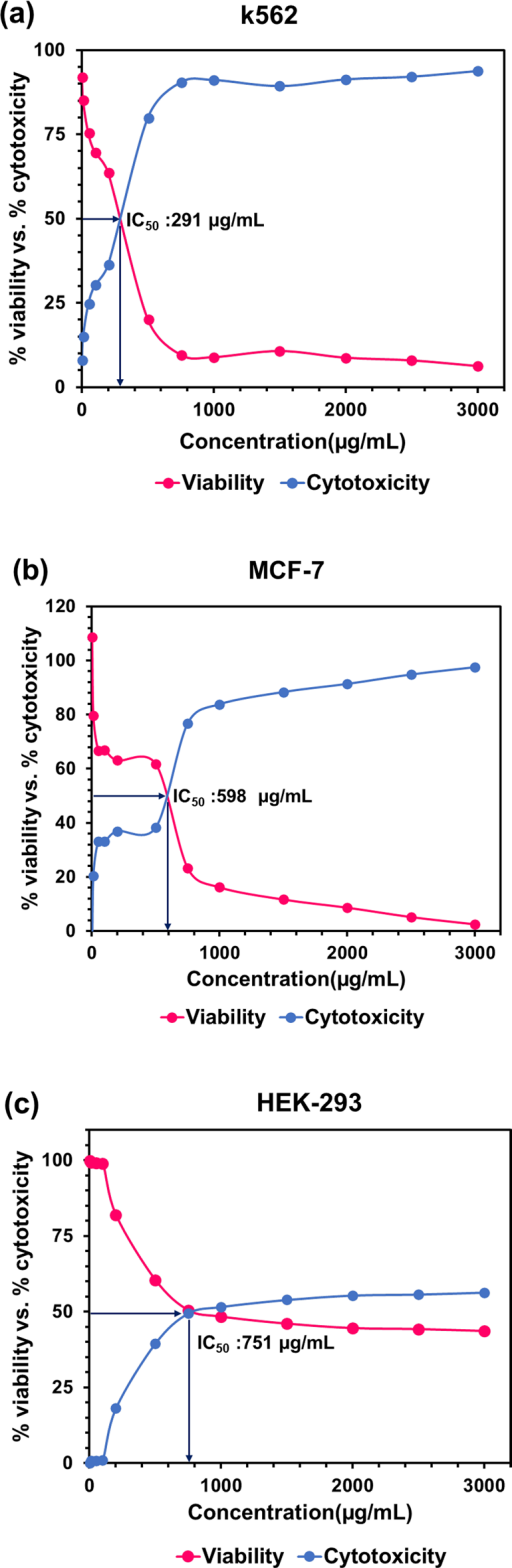
IC_50_ diagrams of (**a**) K-562, (**b**) MCF-7, and (**c**) Hek-293 cell lines treated with *GLME*.

According to the results of the MTT assay, the viability of normal Hek-293 and PDL cells treated with *GLME* at the highest concentration was 43.63 ± 0.4% and 57.81 ± 3.42%, respectively. Viability increased with decreasing concentration, and at a concentration of 1 µg mL^-1^, viability was found to be 99.88 ± 0.335% and 87.1 ± 0.45%, respectively. In the vicinity of normal cells, low toxicity of *GLME* was observed. However, when the *GLME* was placed near MCF-7 and K-562 cancer cells, toxicity increased significantly for the two cell lines. The viability of MCF-7 and K-562 cancer cells treated with *GLME* at a concentration of 3000 µg mL^-1^ was only 6.19 ± 0.86% and 2.52 ± 1.38%, respectively. Viability of MCF-7 and K-562 cells was observed to be less than 25% at a concentration of 750 µg mL^-1^, while Hek-293 and PDL normal cell viability at the same concentration was over 50%. The high toxicity of *GLME* near cancer cells is due to the presence of detected anticancer elements in *GLME* (see Fig. 5a). These results suggest that MCF-7 and K-562 cancer cells might display higher sensitivity than normal cells Hek-293 and PDL to *GLME*. MCF-7 and K-562 cell number was reduced, and morphology was affected, in the presence of *GLME* (see Fig. 6). The IC50 calculations determined 751, 598, and 291 µg mL^-1^ for the three *GLME*-treated cell lines Hek-293, MCF-7, and K-562, respectively (see Fig. 7). According to a study by Thameem Fathima et al.^33^, methanolic, ethanolic, and aqueous extracts were tested on two cell lines, a normal Vero cell, and a Hep-G2 cancer cell. Treatment with the methanolic extract of *GLME* at a concentration of 1000 μg mL^-1^ resulted in 80% viability in normal Vero cells and 6.3% in Hep-G2 cancer cells. The obtained outcomes clearly confirmed the outstanding anticancer property of *GLME* on the examined cancer cells.

In this study, two common drugs used in cancer treatment (5-fluorouracil and carboplatin) were used as controls. According to the MTT results, carboplatin is more toxic to Hek-293 cells than 5-fluorouracil, so at a concentration of 200 µg mL^-1^, viability of 51.73 ± 0.85% and 65.64 ± 0.24% was observed, respectively. In contrast, the toxicity of *GLME* at the same concentration was only 20%. The survival rate of both drugs, 5-fluorouracil and carboplatin, on MCF-7 cancer cells at a concentration of 200 µg mL^-1^ was 68.143 ± 0.143% and 72.74 ± 2.26%, respectively, while the toxicity of *GLME* on the MCF-7 cell line was higher and a survival rate of 63.08 ± 3.71% was observed. According to the results, *GLME* is more effective than conventional cancer treatment agents (see Fig. 5b,c).

### Antimicrobial studies

The inhibitory activity of *GLME* against selected bacterial strains (i.e., *Staphylococcus aureus*, *Enterococcus faecalis*, *E. coli*, and *Pseudomonas aeruginosa*) was investigated using the microdilution method. It can be seen that the antibacterial effects increased with increasing concentration in a concentration-dependent manner. The results showed that this product's inhibitory effects against Gram-negative and Gram-positive bacterial strains were not similar. From the data presented in Table 2, this valuable product exhibited higher antibacterial activity against *E. coli* and *Pseudomonas aeruginosa*. The MIC results showed that the *GLME* sample with a 125 µg mL^-1^ concentration had the highest inhibitory activity against *E. coli* and *Pseudomonas aeruginosa* bacteria.

**Table 2:** Performance of the *GLME* and streptomycin against selected microorganisms.

Microorganisms	*GLME*			Streptomycin (positive control)			DMSO (negative control)
	Zone of inhibition (mm, (Mean ± SD)	MIC (µg mL^-1^)	MBC (µg mL^-1^)	Zone of inhibition (mm, (Mean ± SD)	MIC (µg mL^-1^)	MBC (µg mL^-1^)	Zone of inhibition (mm, (mean ± SD), MIC (µL mL^-1^) and MBC (µL mL^-1^)
*E.coli*	44 ± 0.09	125	125	30 ± 0.11	125	125	-
*Pseudomonas aeruginosa*	36 ± 0.1	125	> 125	33 ± 0.32	> 62.5	125	-
*Staphyloccus aureus*	28 ± 0.07	250	250	42 ± 0.64	> 62.5	125	-
*Enterococcus faecalis*	34 ± 0.2	250	> 250	41 ± 0.18	31.25	62.5	-

**Figure 8:**
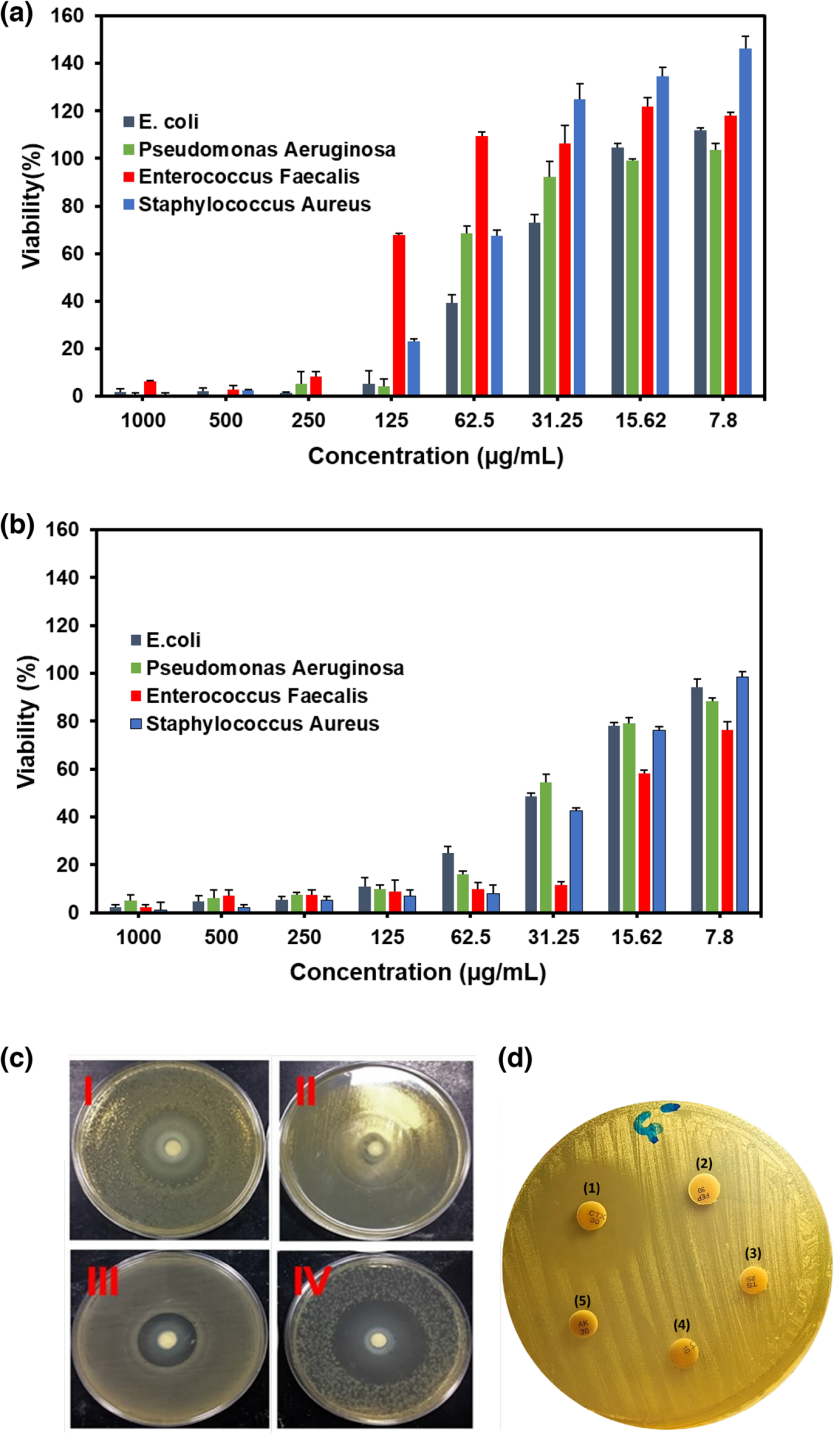
(**a**) Effects of methanolic *GLME* extraction on various microorganism viability percentages in different concentrations (each bar reflects the mean SD (standard deviation) of three independent tests). (**b**) Inhibition zone of *GLME* against (I) *Pseudomonas aeruginosa*, (II) *Enterococcus faecalis*, (III) *Staphylococcus aureus*, and (IV) *Escherichia coli*. (d) Inhibition zone of *Streptomycin* (positive control) at concentrations of (1) 1000 µg mL^-1^and DMSO (negative control) at concentrations of (2) 1000 µL mL^-1^, (3) 250 µL mL^-1^, (4) 31.25 µL mL^-1^ and (5) 7.8 µL mL^-1^ against *Staphylococcus aureus*.

The lethal concentration of the two bacterial strains also did not change. *GLME* had less antibacterial properties against *Staphylococcus aureus* and *Enterococcus faecalis*, so the inhibitory concentration and lethal concentration were 250 µg mL^-1^ (see Fig. 8a). Also, the MIC results of streptomycin as a positive control showed that the highest inhibitory effect of this drug on Enterococcus faecalis was at a concentration of 31.25 µg mL^-1^. DMSO was also used in this research as a negative control in different concentrations from 1000 to 7.8 µL mL^-1^. According to MIC assay, no antibacterial property was observed in any concentration (see Fig. 8b). As expected, the zone of inhibition of *E. coli* and *Pseudomonas aeruginosa* had a larger diameter than the other two strains. According to the results of the *GLME* sample, it had a sizeable antibacterial effect against *E. coli*, and the largest diameter of the zone of inhibition, 44 mm, refers to this bacterium. The commercial antibiotic streptomycin inhibited the growth of all four species (*E.coli, Pseudomonas aeruginosa, Staphylococcus aureus,* and *Enterococcus faecalis*), at a concentration of 1 mg mL^-1^ of the antibiotic with an inhibition zone diameter of 30 ± 0.11, 33 ± 0.32, 42 ± 0.64, 41 ± 0.18 mm, while streptomycin with an inhibition zone of 30 ± 0.11 mm was effective against *E.coli*, *GLME* with an inhibition zone of 44 ± 0.09 mm was more effective than streptomycin. *GLME* was measured to possess bactericidal and bacteriostatic activities against Gram-negative and Gram-positive strains, mainly due to the polysaccharide components in its structure. Figure 8c shows a view of the disk diffusion method after exposure to four different microorganisms. Also Fig. 8d shows a view of Inhibition zone of Streptomycin (positive control) at concentrations of 1000 μg mL^-1^ and DMSO (negative control) at concentrations of 1000, 250, 31.25, and 7.8 µL mL^-1^ against *Staphylococcus aureus*. According to the results, DMSO had no antibacterial activity at all concentrations.

The mean zones of inhibition in the disc diffusion (DD) method, with a disc diameter of 6 mm, were measured in millimeters. The minimum inhibitory concentration (MIC) and minimum bactericidal concentration (MBC) were measured in mg mL^-1^. The initial doses of both medications were identical (1 mg mL^-1^ in 1:1 dilution).

## Discussion

*GLME* is a type of mushroom that is abundant in bioactive compounds. These compounds include nucleosides, fatty acids, proteins, amino acids, peptides, alkaloids, steroids, triterpenoids, enzymes, and polysaccharides^40,41^. The *GLME* polysaccharides have immunomodulatory properties that activate the expression of cytokines associated with the inflammatory response and antitumor activity. These cytokines include interleukin-1, interleukin-6, tumor necrosis factor-α, and interferon-γ^42^. Although some authors suggest that the polysaccharides of *GLME* have a direct cytotoxic effect on cancer cells, it is generally believed that their anticancer functions are closely related to their immunostimulatory effects. Raj et al.^43^, reported that polysaccharides and triterpenoids extracted from *GLME* have antiproliferative effects on cancer cells. In addition to polysaccharides, extracts of *GLME* may also contain other phytochemicals responsible for their cytotoxic effects on cancer cells. According to Veljovi et al., an ethanolic Ganoderma extract containing phenolic compounds was found to have an antiproliferative effect on a number of cancer cell lines, including A549 (lung cancer cell line), HeLa (cervical cancer cell line), EA.hy 926 (permanent human cell line generated by fusion of human umbilical vein endothelial cells (HUVEC) with human lung adenocarcinoma epithelial cells (A549)), and LS174 (colon cancer cell line)^44^. Previous research shows that *GLME* extract has extraordinary anticancer effects on cancer cell lines. In the present study, the MTT assay of *GLME* was investigated near two normal cell lines (PDL and Hek-293) and two cancer cell lines (MCF-7 and K-562). The *GLME* had significant toxicity against MCF-7 and K-562 cancer cells. The viability of MCF -7 and K-562 cells treated with *GLME* at a concentration of 750 µg mL^-1^ was found to be 23.22 ± 2.9% and 9.55 ± 0.847%, respectively. In contrast, for the Hek-293 and PDL cell lines, the toxicity of *GLME* at a concentration of 750 µg mL^-1^ was only 50% and 26.5%, respectively. The significant toxicity of *GLME* in the present study may be due to the presence of compounds such as hexanoic acid, pentadecanoic acid, 14-methyl ester, and (Z, Z)-9,12-octadecadienoic acid methyl ester. These compounds were observed in GC/MS analysis with a high percentage.

Numerous substances have antimicrobial effects that affect the bacterial cytoplasmic membrane, including terpenes, polysaccharides, and lectins^45,46^. According to Gao et al., *GLME* and other Ganoderma species have been utilized to treat a variety of bacterial infections, frequently in conjunction with chemotherapy drugs^47^. The bioactive component of *GLME* responsible for its antibacterial action has been discovered to be polysaccharide-based. Sena et al.^48^ found that the *GLME* derivative methyl australite had the least inhibition zone for *Bacillus* species and the highest antibacterial activity against *E. coli* and *P. aeruginosa*. The methanol extract of *GLME* has been found to exhibit remarkable antibacterial activity against various bacterial species, including *Salmonella* species, *E. coli*, and *B. subtilis*, according to Sheena et al.^49^. Yoon et al.^50^ reported that an aqueous extract of *GLME* had a significantly high minimum inhibitory concentration (MIC) against *B. subtilis* (3.5 mg mL^-1^) and *Bacillus* species (3.5 mg mL^-1^). Heleno et al.^51^ found strong potential for activity against *S. aureus* and *E. coli* in the phenolic extract of Portuguese *GLME*, with MICs and MBCs of 0.025 and 0.35 mg mL^-1^ and 0.035 and 0.75 mg mL^-1^, respectively. The extract has more potent antibacterial effects than the reference materials (ampicillin and streptomycin).

In the current study, the antibacterial activity of *GLME* was examined against four bacteria utilizing the disc diffusion method (DD), MIC, MBC, and inhibition zone assays. The MIC results showed that *GLME* had the highest antibacterial activity against *E.coli* and *Pseudomonas aeruginosa* bacteria at a concentration of 125 µg mL^-1^. The zone of inhibition of the above bacteria was 44 ± 0.09 and 36 ± 0.1 mm, respectively. The exceptional antibacterial property of *GLME* is evident. Numerous organisms are resistant to antibiotics, and multi-resistant organisms pose a significant challenge to managing infectious diseases.

Consequently, antimicrobials derived from fungi have garnered much attention in recent years. The present study shows that *GLME* could combat various diseases caused by pathogenic microorganisms^52,53^. Overall, our data suggest that *GLME* may be a novel strategy for treating cancer and bacterial infections because it contains a wide range of anticancer and antibacterial compounds.

## Conclusion

In summary, the anticancer and antibacterial activities of *GLME* were comparatively measured in several cell lines and bacteria using the MTT and MIC assays. This work is motivated by research studies seeking pharmacological products to treat chronic and acute diseases, and further resources and studies are needed to investigate the adverse effects and toxicity of such products. The *GLME* inhibited cancer and bacterial growth according to the anticancer and antibacterial activity tests at the lowest dilutions. In the future, molecular studies and related techniques can be used to investigate the active components of *GLME* that play a role in controlling and preventing the proliferation of various cancer cells and infections caused by common microorganisms. The use of *GLME* may open a new therapeutic opportunity for treating and managing blood and breast cancer during chemotherapy, especially considering its potent antibacterial effect, which may lead to its use as a new therapeutic agent in cancer treatment.

## Data Availability

All data generated or analyzed during this study are included in this published article.

## Abbreviations

XRD: X-ray diffraction
FESEM: Field emission scanning electron microscopy
FTIR: Fourier-transform infrared spectroscopy
UV: Ultraviolet light
HPLC: High-performance liquid chromatography
GL: *Ganoderma lucidum*
GLME: *Ganoderma lucidum* Methanolic extract
MIC: Minimum inhibitory concentrations
MBC: Minimum bactericidal concentrations
EDX: Energy dispersive spectroscopy
CFU: Colony forming units
DMEM: Dulbecco’s modified eagle medium
OD: Optical density
MTT: (3-(4,5 Dimethylthiazol-2-yl)-2,5-diphenyltetrazolium)
DMSO: Dimethyl sulfoxide
